# A framework for blood biomarker discovery in MS

**DOI:** 10.1177/13524585261432045

**Published:** 2026-03-30

**Authors:** Emma C Tallantyre, Sean Apap Mangion, Charlotte E Teunissen

**Affiliations:** Division of Psychological Medicine and Clinical Neuroscience, Cardiff University, Cardiff, UK; Queen Square Multiple Sclerosis Centre, Department of Neuroinflammation, Institute of Neurology, University College London, London, UK; Neurochemistry Laboratory, Department of Laboratory Medicine, Amsterdam Neuroscience, Neurodegeneration, Amsterdam UMC, VU University Amsterdam, The Netherlands

**Keywords:** Multiple sclerosis, biomarker, clinical trials

## Abstract

Multiple sclerosis (MS) biomarkers hold potential for improving pathophysiological understanding, diagnostics, prognostication, and treatment personalisation. Technological advances now permit detection of blood biomarkers at previously prohibitively low concentrations. Concentrations of several candidate biomarkers have been shown to correlate between blood and cerebrospinal fluid (CSF). The development of ‘-omics’ panel-based technologies now facilitates the discovery of collections of molecules, the interdependency of which may better reflect disease heterogeneity. Neurofilament light (NfL) is the clearest example of the success of a biomarker entering the realm of clinical practice in MS, with promising developments in others such as glial fibrillary acid protein (GFAP). However, integration of new biomarkers requires standardisation of discovery and validation across the various available technologies, along with demonstration of clinical utility, to gain regulatory approval and widespread adoption. In this review, focused on blood-based biomarkers, we expand on the above discoveries, developments and trajectories, to provide a framework for understanding this crucial body of work.

## Introduction

Biomarker discovery in multiple sclerosis (MS) provides an important opportunity to grow our understanding and refine our predictions in a complex and heterogeneous disease. The study of biomarkers in cerebrospinal fluid (CSF) has the benefit of close proximity to central nervous system (CNS) pathology, but the ability to detect candidate biomarkers in blood at low concentration, and the growing use of multi-omic technologies to reflect wider aspects of disease pathways, mean that blood biomarkers hold promise to meet urgent unmet needs in MS including improved diagnosis, prognostication, monitoring of disease activity, prediction and assessment of treatment response, and unravelling of disease pathophysiology.

Despite significant progress, the implementation of blood biomarkers into clinical practice remains challenging due to MS heterogeneity, the need for robust validation in diverse cohorts, and the need for standardisation across centres and investigative methodologies. It is possible that combination biomarkers or multi-modal approaches will be required to fully capture the nuanced pathology of MS.

## Potential utility

The use of fluid biomarkers in the diagnosis is not new; oligoclonal bands have been included in international diagnostic criteria for MS for over 40 years. However, blood biomarkers could provide a less invasive way of providing paraclinical evidence of diagnosis in the future.^1,2^ Likewise, blood biomarkers may have future utility in stratifying risk of MS in those in a prodromal phase or in an at-risk population.^3^ Blood biomarkers are already in use to enhance the safety of disease-modifying therapies (DMTs) in MS (e.g. John Cunningham Virus (JCV) index in people treated with natalizumab). Perhaps the greatest unmet need, however, given the heterogeneous disability trajectories of people with MS, is blood biomarkers that can predict and reflect disease activity at onset and throughout the disease course, including biomarkers indicative of treatment response. Such biomarkers might inform the type and intensity of treatment, particularly if they could help disentangle the relative contributions of pathological processes including focal and diffuse inflammation, chronic demyelination and neuro-axonal degeneration. Pathologically specific biomarkers also hold promise as surrogate outcome measures in early-phase clinical trials, interim outcome measures in multi-arm multi-stage trials, remote outcomes in hybrid trials or the opportunity for trial enrichment – reducing trial durations and participant numbers.

## Candidate blood biomarkers

Neurofilament light (NfL) is the blood biomarker which has been subject to the most investigation in MS. Studies have shown serum neurofilament light (sNfL) to reflect contemporary inflammatory disease activity^4–6^ and to predict medium- and long-term disability and magnetic resonance imaging (MRI) outcomes such as brain atrophy.^7–9^ NfL has been shown to change with age, body mass index (BMI) and renal function in control populations, but normative values have been developed to aid interpretation.^5^ Consensus emerging on the value of serum NfL points towards a relatively non-invasive and inexpensive way to detect subclinical disease activity in people with MS, especially those on DMT.^10^ While it seems unlikely to completely replace MRI, which offers additional benefits of lesion topography and biological specificity, sNfL could offer a triage test to perform an MRI scan^11^ and has already been demonstrated to align with clinical outcomes in clinical trials.^7^

Glial fibrillary acidic protein (GFAP) is a marker of astrocyte reactivity and gliosis, a prominent feature in MS progression. Serum glial fibrillary acidic protein (sGFAP) has been shown to be higher in people with progressive versus relapsing disease courses of MS and to be predictive of disability progression and brain atrophy.^12–16^ GFAP was identified as the plasma biomarker most predictive of progression independent of relapses (PIRA) out of 322 candidate proteins in two multiplex NUcleic Acid-Linked Immuno-Sandwich Assay (NULISA) panels.^17^ Comparison of sNfL and sGFAP suggests that high sNfL is most predictive of relapse-associated worsening (RAW), which can be mitigated by DMT, whereas high sGFAP is more reflective of PIRA.^16,18^ Likewise, there is evidence that sNfL is significantly reduced by both high- and low-efficacy DMTs, whereas sGFAP is less affected by immunomodulatory DMT.^19,20^ Surprisingly, although sGFAP is postulated to be a marker of MS disability progression, a large cohort of people with progressive MS found that sGFAP predicted EDSS progression in people with SP but not PPMS.^21^

Chitinase-3-like-1 (Chi3L1; also known as YKL-40) is a protein produced by cells including macrophages and astrocytes, which has been linked to neuroinflammatory processes and reactive gliosis. Chi3L1 in the CSF has been shown to associate with conversion from clinically isolated syndrome (CIS) to MS,^22^ progressive versus relapsing disease course^23^ and exposure to/response to high-efficacy DMT.^24,25^ Evidence supporting a role of Chi3L1 in MS pathogenesis includes the finding of astrocytes expressing Chi3L1 at the edge of chronically active MS lesions.^26^ Chi3L1 levels appear to correlate between CSF and plasma samples.^27^ Serum Chi3L1 (sChi3L1) emerged as one of four serum markers to predict long-term disability outcomes in a large unbiased proteomics approach,^27^ and sChi3L1 was found to be a stronger predictor of EDSS change than sNfL or sGFAP in two independent MS cohorts.^28,29^ sChi3L1 has also been shown to reflect response to beta-interferon in people with MS.^30^

CXCL13 is a B-cell chemoattractant, involved in the formation of lymphoid follicles, and has also emerged as a candidate prognostic marker in MS. Although serum CXCL13 (sCXCL13) levels can be increased by infectious and neoplastic processes,^31^ levels have been shown to associate with neurological involvement in systemic autoimmune conditions,^32^ and change in sCXCL13 from baseline to 12 months was predictive of treatment response (evidence of relapse, EDSS progression or active lesions) in people with MS receiving teriflunomide.^33^

Complement forms an important link between innate and adaptive immune systems and has been postulated to contribute to MS pathogenesis. Pathological and functional studies have demonstrated complement regulators and complement activation products within chronically active lesions^34^ and biological fluids of people with MS. Complement can be susceptible to ex vivo activation, so strict practices of sampling and pre-analytical sample handling are vital.^35^ Several studies have demonstrated an association between serum/plasma concentrations of complement proteins (including C1q, C3, C4, C5, C9, C1-inhibitor, Factor H and Factor I) in the blood and MS relapse activity or disability trajectory.^29,36–38^

There is evidence that using combinations of blood biomarkers could offer greater utility and highlight different pathological processes than single biomarkers. For instance, the combination of sGFAP and sNfL has been shown to provide complementary information and better predictions than either alone.^14,16,18^ Several groups have shown that combining a panel of blood biomarkers improved prediction of diagnosis, relapse or disability progression compared to any single blood biomarker used in isolation.^29,39,40^

## Newer approaches to biomarker discovery

New and emerging approaches to blood biomarker discovery for MS will benefit from several key areas of innovation. First, advanced analytical platforms now enable detection of low concentrations of disease-relevant molecules within the blood. Second, multi-modal strategies that integrate diverse biological data (e.g. -omics or studies combining advanced imaging and blood biomarkers) hold potential to shed new light on disease pathogenesis. Finally, improvements in computational analysis (Artificial Intelligence and Machine Learning) are likely to continue to add value in identifying patterns among the noise generated by large, multi-dimensional datasets.^41^

‘Omics’ investigations include genomics, transcriptomics, epigenetics, proteomics and metabolomics. Platforms often provide high throughput and require small sample volumes, offering an opportunity for multi-dimensional studies of complex diseases. Traditional hypothesis-driven approaches to science explore molecules known to be relevant, based on existing understanding of disease mechanism. On the contrary, -omics studies are usually discovery-driven, using datasets to explore alterations in the abundance or expression of many molecules in cases versus controls, or in people at different phases of a disease.

Early proteomics approaches, using gel electrophoresis to resolve and visualise many individual proteins on a single gel, have largely been replaced with newer technologies including advanced mass spectrometry and affinity-based methods such as Olink, NULISA and SomaScan. Mass spectrometry, which is achieved by digesting proteins into smaller peptide fragments, is unbiased and can be used to deduce post-translational modifications (e.g. phosphorylation and glycosylation). Mass spectrometry has historically been mainly sensitive to highly abundant proteins, with limited detection of disease-relevant proteins in MS such as cytokines and chemokines, but newer methods offer improved sensitivity. Affinity-based methods use a multiplex approach, enabling hundreds or thousands of proteins from a particular library to be measured concurrently. The Olink and NULISA platforms, affinity-based proximity extension approaches, use two different antibodies, each tagged with a unique DNA oligonucleotide, to recognise each target protein. In the proximity extension technology, when the antibodies both bind, the two DNA oligonucleotides are brought into proximity so that they anneal, creating a new, unique DNA molecule that corresponds to that target protein, which can be quantified using quantitative polymerase chain reaction (qPCR). These newer proteomic approaches are somewhat complementary and generally have trade-offs between proteome coverage versus precision and absolute quantification.

Early proteomics studies mainly used CSF, but some candidate markers are subsequently validated as being relevant in the blood. For example, CSF mass spectrometry studies first uncovered Chi3L1 as a candidate biomarker in MS,^42^ and subsequently demonstrated its relevance in predicting treatment response.^43^ Åkesson et al.^27^ used proximity extension assay (PEA) proteomics in both CSF and blood samples. They identified 52 out of 1463 proteins with differential expression in CSF from people with MS versus controls, of which 23 overlapped with CSF findings from an independent validation cohort, and 11 (CXCL13, LTA, FCN2, ICAM3, LY9, SLAMF7, TYMP, CHI3L1, FYB1, TNFRSF1B, NfL) predicted long-term disability outcomes. However, the same data-driven approach using blood samples did not uncover any predictive biomarkers, and of the 11 candidates identified in CSF, NfL was the only protein in plasma samples to significantly predict disability outcomes.^27^ Gross et al. used white cell immunophenotyping in a large, multi-centre cohort to identify three discrete immune endophenotypes of MS, 2 of which had distinctive clinical and treatment-response characteristics. However, proximity extension proteomic analysis only identified 3 out of 1450 proteins that aligned with these disease categories.^44^

Other groups have used unbiased proteomic screening approaches to reveal novel peptides or proteins, that still require subsequent validation using more sensitive analytical approaches if they are to be taken forward as candidate predictive markers for clinical practice.^45–49^ But the unbiased, data-driven approach may also hold promise for the generation of knowledge about MS pathogenesis. The network analysis performed by Åkesson et al. showed many more interactions between candidate proteins than would be expected by chance, indicating shared functionality, lending weight to the relevance of co-stimulatory processes that allow B-cells to maintain activation of pathogenic T-cells, including a potential role of Epstein–Barr virus (EBV). The screening approach and network analysis also uncovered other candidate proteins that were not covered by the proteomics platform but appeared in the same densely connected network.^27^ Lewin et al.^50^ used findings from unbiased mass spectrometry of blood samples from people with MS, and associated brain atrophy data, to hypothesise that chronic, low-grade haemolysis could be partially responsible for the hallmark iron deposition seen in people with advanced disability. Lin et al.^51^ demonstrated the potential utility of combing -omics data by integrating proteomics data from blood and brain samples with genomic susceptibility data to identify pathways with potential for therapeutic targeting.

PEAs can also be used to combination biomarker panels that try to overcome the challenges of developing multiplex panels (designing an assay that overcomes the fact that each biomarker requires distinct conditions for optimal quantification using traditional methods). A multiple sclerosis disease activity (MSDA) panel has been developed using Olink PEA technology, where selection of 18 to 20 proteins was made according to expected relevance in MS, feasibility experiments exploring the relationship of >1400 proteins with markers of MS disease activity, and technical validaton.^52,53^ Clinical validation of the MSDA panel in a cohort of 614 people with MS enriched for those with enhancing lesions (~40%) showed an area under the receiver operator characteristic curve (AUC) of 0.73 for NfL and 0.78 for the multi-marker panel.^54^ Further validation showed improvement of disability outcome prediction from AUC = 0.85 using clinical features, to AUC = 0.91 using combined clinical and multiplex panel data, in which the best-performing protein markers were CUB-domain-containing protein 1 (CDCP1), interleukin-12 subunit beta (IL-12B), NfL and protogenin (PRTG).^55^

Metabolomics is the study of small molecules (<1500 Da) aimed at identifying metabolic pathways that are perturbed in disease states. Metabolomics are likely to be most helpful in uncovering the contribution of various metabolic pathways in disease pathophysiology, but it is also possible that distinct metabolic signatures may become useful as biomarkers of certain disease states (e.g. MS vs. other neuroinflammatory conditions, relapse vs. progression, markers of treatment response). Metabolomics includes the detection of molecules beyond proteins (e.g. lipids, nucleotides) and can consider the interaction between metabolic disruption and disease expression and the effect of environmental factors on disease state. Discrepancies between studies in MS may arise due to variation in pre-analytical methods (sample collection and preparation) and analytics (both laboratory and statistical). Metabolomic studies of serum that target-specific pathways have demonstrated a profile of metabolites reflecting dysfunctional energy metabolism,^56^ and excessive activation of the arachidonic acid pathway in perpetuating innate inflammation,^57^ in people with progressive MS. Likewise, targeted studies have revealed that metabolites derived from gut microbiota appear to be protective against the risk of MS and the risk of disability in children.^58^ Global untargeted metabolomics demonstrates alterations of many metabolites in numerous pathways, which can provide a challenge in distilling out translational messages on mechanism but has shown some proof of concept on how blood-based metabolomics approach could facilitate the identification of novel therapeutic strategies.^59,60^ The value of metabolomics is likely to be felt more in the research setting (exploring mechanisms and pathways) rather than in identifying clinical biomarkers. Barriers to translating metabolite panels to the MS clinic include the potential need to standardise pre-analytical sample handling, account for environmental confounders and standardise data arising from a range of platforms. Metabolite panels described to date have diagnostic/predictive accuracy below the level required for clinical practice.^61^

Extracellular vesicles (ECVs) are particles enclosed by a lipid bilayer, which are actively secreted by a range of cell types into the extracellular space, possibly facilitating cell-to-cell communication and transfer of cellular constituents over long distances. ECV cargo includes lipids, proteins and micro-RNAs (miRNAs; noncoding, single-stranded RNA segments that control gene expression and protein synthesis). The ability of ECVs to traverse the blood–brain barrier provides a potentially valuable opportunity to derive information on inaccessible tissues using peripheral blood samples, leading to the description of a ‘liquid biopsy’. Mrad et al.^62^ showed that exosomes from 30 people with relapsing-remitting multiple sclerosis (RRMS) (especially those with concurrent disease activity) expressed higher EBV proteins than exosomes from 15 healthy controls and that MS exosomes activated macrophages more avidly than controls. Likewise, Galazka et al.^63^ found that ECVs derived from peripheral blood of people with MS contained CNS myelin proteins and more avidly induced the proliferation of myelin-reactive T-cells than control exosomes. Ebrahimkhani et al.^64^ sequenced miRNAs derived from serum ECVs, identifying 11 differentially expressed miRNAs between 14 people with relapsing-remitting MS and 11 people with progressive MS, subsequently validating that three of those miRNAs (miR-223-3p, miR-485-3p, miR-30b-5p) could distinguish a second sample of 11 people with progressive MS from the original RRMS cohort. The miRNAs showed relevance to neurotrophin signalling, T-cell receptor signalling and focal adhesion pathways. Selmaj et al. found lower expression of four miRNAs in people with RRMS (especially people with concurrent disease activity) versus controls. Based on pathway analysis, they hypothesised that lower expression of these ECV miRNAs in people with MS may result in reduced function of STAT3, which is a key controller of the autoimmune T helper cell differentiation.^65^ The study of ECVs provides an opportunity to study cell-to-cell communication in MS. Exosome miRNA may have utility as disease biomarkers, but studying ECVs presents considerable technical challenges, including a lack of standardisation of methods, for example, extraction and purification, and variations in ECV cargo according to sample type.

A strength of the -omics approach is the identification of molecules that have not been formerly related to MS, but the approach relies upon good phenotyping and advanced statistics to distil out the most relevant candidates, all of which need subsequent validation in independent cohorts. Given the complexity of MS, the integration of different -omics datasets holds potential for further insights into disease pathways and uncovering new biomarkers, for example, by integrating indicators of altered gene expression and metabolic dysfunction.

## Pathway to implementation

**Figure 1.**
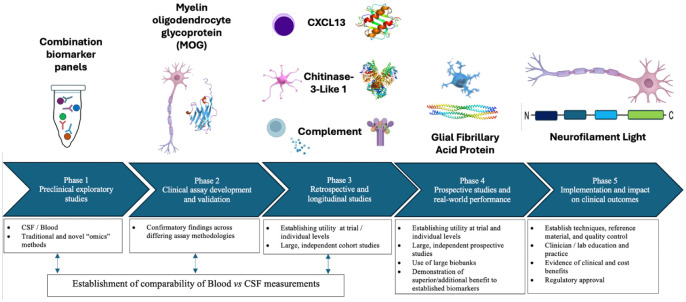
Pathway of blood biomarker development in MS, illustrating the current position of some candidate markers. This figure was adapted from Teunissen et al. Lancet Neurol 2022; 21: 66–77 and created using images from the following websites: ChatGPT: neuron; Wikimedia: CXCL13 (File: Protein_CXCL1_PDB_1mgs.png), GFAP (File: GFAP_protein.png), Lymphocyte (File:Small_lymphocyte_(4).svg), Astrocyte and Glia (Blausen.com staff (2014). ‘Medical gallery of Blausen Medical 2014’. WikiJournal of Medicine 1 (2). DOI:10.15347/wjm/2014.010. ISSN 2002-4436), Cell and complement (Nature CC BY: https://www.nature.com/articles/s41392-023-01675-2), MOG (https://www.ebi.ac.uk/pdbe/entry/pdb/1pko).

The early discovery phase of biomarkers focuses on the technical performance of the assay including reproducibility, repeatability and limits of detection. Initial clinical validation provides evidence that a biomarker is sensitive and accurate for a desired clinical outcome measure on an individual basis (not just a group-wise effect). Even when these steps have highlighted a biomarker with potential utility, standardisation is required including pre-analytical pathways (e.g. specimen collection and processing), provision of normative values (including any required corrections for age, sex or other factors) and a rigorous approach to calibration is required including standardisation and harmonisation of different assays and platforms. Figure 1 shows the pathway to development, illustrated with some candidate markers.

To achieve regulatory approval and widespread adoption, biomarkers should demonstrate either improved predictions versus existing tools or additional value, for example, convenience. Studies suggest that the addition of blood biomarkers (sGFAP and/or sNfL) only modestly improves predictions of MS outcome versus models using clinical and radiological measures traditionally used in the clinic.^55,66^ However, the value of blood biomarkers may be saving the need for more costly or invasive tests. Serum neurofilament is the blood biomarker furthest along this pathway to implementation, with random access automatation and approval by Food and Drug Administration (FDA, USA)/*Conformité Européenne* In Vitro Diagnostic Medical Devices (CE-IVDR, Europe) of multiple platforms available or pending (Elecsys, Simoa, Attelica, Lumipulse).

Some barriers remain before biomarkers can be incorporated into widespread clinical practice. International consensus on how to incorporate blood biomarkers into existing clinical practice might include how they would complement/replace MRI and which thresholds should trigger treatment escalation. Clinicians may require further evidence on cost-effectiveness to confirm that integration of biomarkers into practice improves disease outcomes.

## Future directions and challenges

The ability to combine less invasive sampling (blood vs. CSF) with laboratory techniques that simultaneously measure large panels of biomarkers holds great promise. Studies combining radiological, biological and detailed phenotyping data (e.g. PIRA vs. RAW) are most likely to further understanding of which pathophysiological process is captured by which blood biomarker. Close collaboration between groups experienced in biobanking (e.g. via the BioMS network – bioms-eu.com), collation and sharing of data on sample collections will also aid identification and robust validation of new biomarkers. Fully realising the clinical utility of blood biomarkers will require agreement on universally accepted cut-off values and standardised reference ranges, including any correction required for variables including age, sex and BMI. It is possible that in the future, by multiplexing assays, combinations of the most promising markers could be run on a single chip.

In conclusion, the study of blood biomarkers is already helping to unravel the complex pathophysiology of MS and to identify candidate markers for diagnosis, prediction and prognosis with promise for clinical utility. The path to clinical implementation is arduous, requiring collaborative efforts between scientists, clinicians, industry and regulatory bodies to address technical, systemic and patient-centric challenges. Combinations of blood biomarkers, including the possibility of multi-omic combinations, seem most likely to convey the complex interplay of processes that culminate in the heterogeneous outcomes typical of MS.
